# A probabilistic approach for assessing the mechanical performance of intertrochanteric fracture stabilized with proximal femoral nail antirotation

**DOI:** 10.1371/journal.pone.0299996

**Published:** 2024-04-11

**Authors:** Zhiqi Zhu, Yi Yang, Lunjian Li, Shuang Jie Zhu, Lihai Zhang

**Affiliations:** 1 Department of Orthopedics, Longgang District People’s Hospital of Shenzhen, Guangdong, P. R. China; 2 Department of Infrastructure Engineering, The University of Melbourne, Victoria, Australia; 3 Department of Mechanical and Product Design Engineering, Swinburne University of Technology, Victoria, Australia; Indian Institute of Technology Guwahati, INDIA

## Abstract

Maintaining post-operative mechanical stability is crucial for successfully healing intertrochanteric fractures treated with the Proximal Femoral Nail Antirotation (PFNA) system. This stability is primarily dependent on the bone mineral density (BMD) and strain on the fracture. Current PFNA failure analyses often overlook the uncertainties related to BMD and body weight (BW). Therefore, this study aimed to develop a probabilistic model using finite element modeling and engineering reliability analysis to assess the post-operative performance of PFNA under various physiological loading conditions. The model predictions were validated through a series of experimental test. The results revealed a negative nonlinear relationship between the BMD and compressive strain. Conversely, the BW was positively and linearly correlated with the compressive strain. Importantly, the compressive strain was more sensitive to BW than to BMD when the BMD exceeded 0.6 g/cm^3^. Potential trabecular bone compression failure is also indicated if BMD is equal to or below 0.15 g/cm^3^ and BW increases to approximately 2.5 times the normal or higher. This study emphasizes that variations in the BMD significantly affect the probability of failure of a PFNA system. Thus, careful planning of post-operative physical therapy is essential. For patients aged > 50 years restrictions on high-intensity activities are advised, while limiting strenuous movements is recommended for those aged > 65 years.

## Introduction

Intertrochanteric fractures which are defined as extracapsular fractures of the proximal femur occurring in between the greater and lesser trochanters is the most common type of hip fracture [1]. Of all the types of hip fractures, intertrochanteric fractures have significant implications for societal health and financial burden [2]. Age-related fractures are associated with increased morbidity and mortality rates. Alarming statistics indicate that approximately 30% of elderly patients die within the first year after an intertrochanteric fracture [3]. Moreover, 10–20% of patients experience functional disabilities and subsequent loss of autonomy [4–6].

Surgical management of intertrochanteric fractures requires secure fixation to enable early mobility and reduce the risk of complications. Options for surgical treatment of intertrochanteric fractures include intramedullary (*e*.*g*., Gamma nail) [7] and extramedullary fixations (*e*.*g*., dynamic hip screw) [7]. Nowadays, Proximal Femoral Nail Antirotation (PFNA), an intramedullary implant, becomes increasingly popular for treating intertrochanteric fractures [8, 9]. This is largely due to their exceptional biomechanical stability [10] and elevated resistance to cut-out [10] which is a phenomenon characterized by the upward migration and protrusion of the screw or blade owing to the ongoing collapse of the femoral head into a varus position [11]. A comprehensive multicenter clinical study evaluating the use of PFNA in routine practice indicated that the post-operative complication rate associated with the bone-implant structure was approximately 6.3%, with the cut-out representing approximately 22% of these instances [12]. Both numerical and clinical studies have identified cut-out as a multifactorial event associated with multiple factors, such as reduction quality of intertrochanteric fracture, implant design, and lag screw position [13, 14].

Given the high incidence of cut-outs in proximal femoral fractures, it is crucial to investigate the factors that contribute to this risk. Cut-out initiation is reportedly associated with elevated strain levels in the trabecular bone [15]. The strain induced by loading is dependent on the elastic modulus of the trabecular bone, which in turn is influenced by the apparent bone mineral density [16]. Numerous experimental studies have suggested a power-law-based correlation between elastic modulus and apparent bone mineral density [17–19]. Moreover, the intensity of post-operative physical activity can significantly affect the strain experienced by the fractured bone. Excessive loading may result in strains exceeding the yield or ultimate limits, whereas underloading may create an unfavorable biomechanical environment for fracture healing [20, 21]. Until now, assessment of bone-implant construct failures under post-operative mechanical stress has primarily relied on deterministic methods using finite element analysis (FEA) [22–24]. However, these studies invariably encounter uncertainties associated with the input variables [20, 25]. For instance, different experimental methodologies can potentially result in significant variance in the elastic modulus of trabecular bone [17]. Numerous mathematical relationships have been proposed to establish the connection between bone mineral density and elastic modulus in the proximal femur [18, 26–30]. While the power law relationship (*E*_*t*_ ∝ *ρ*^*a*^) is a commonly accepted expression to represent modulus in terms of apparent bone mineral density; the power term "a" ranges from 1.40 to 7.4 [18, 26–30], leading to uncertainties in selecting material properties for FEA. Furthermore, patients with bone fractures often struggle to adhere to the weight-bearing guidelines provided by healthcare providers, resulting in uncertain loading conditions on the fractured [31]. Consequently, the performance of the bone-implant system may not be accurately represented, owing to insufficient consideration of variabilities and uncertainties in the input parameters.

In contrast, probability-based analysis offers a valuable approach by integrating statistical data of the inputs, effectively incorporating the effects of uncertainty and variability associated with these parameters into the outcomes. This method, provides insights into the interconnected influences of multiple inputs [32]. While probabilistic studies have traditionally involved assessing the structural reliability of infrastructure [33–36], their application in biomechanics emerged in evaluating the reliability of existing orthopedic constructs and designing new orthopedic systems [37–41]. For instance, Kayabasi and Ekici investigated the effects of design parameters of cemented hip prosthesis on fatigue failure based on probabilistic finite element modelling [39]. Similarly, Mehrez and Browne developed a finite element model in conjunction with Monte Carlo simulation and first-order reliability method for assessing the structural integrity of hip replacement constructs [40].

**Fig 1:**
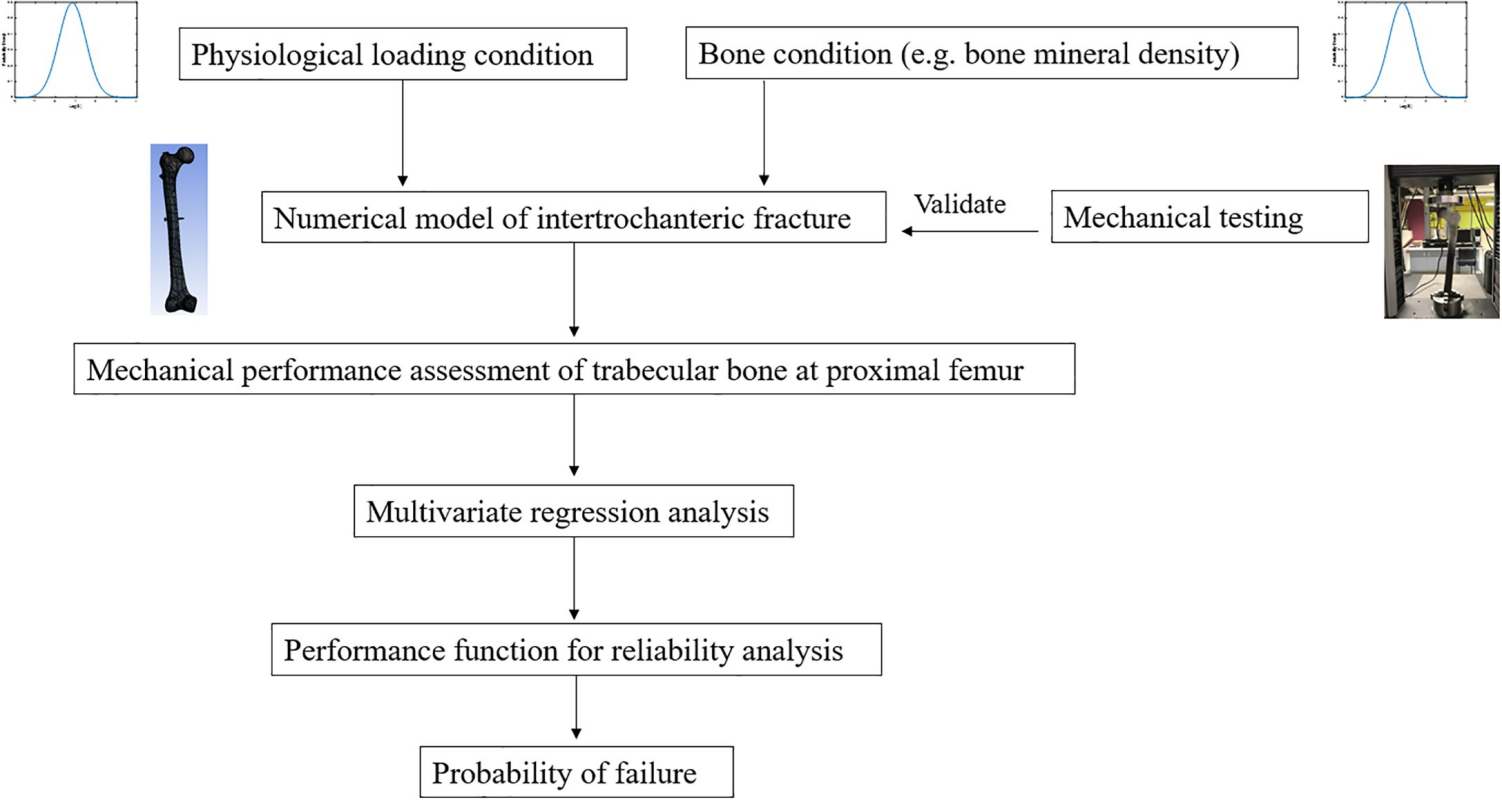
Schematic diagram showing the methodology used in this study.

In this study, we aimed to and successfully developed a reliability-based model specifically designed to evaluate the likelihood of failures in intertrochanteric fractures during the early stages of healing. These fractures were stabilized using PFNA, and the assessment was largely determined by the apparent BMD and physiological loading (Fig 1). Building upon our previous research that focused on uncertainty analysis in tibial fracture healing [20, 25], the refined model may provide actionable guidance for post-operative weight-bearing exercises. These recommendations consider the nuances of everyday activities and are tailored to specific age groups. PFNA for intertrochanteric fractures poses several challenges, particularly cut-out or cut-through. The present research represents a first step towards improving implant design, surgical techniques, and patient management to tackle these challenges.

## Materials and methods

### Mechanical testing of composite femurs stabilized by PFNA

**Fig 2:**
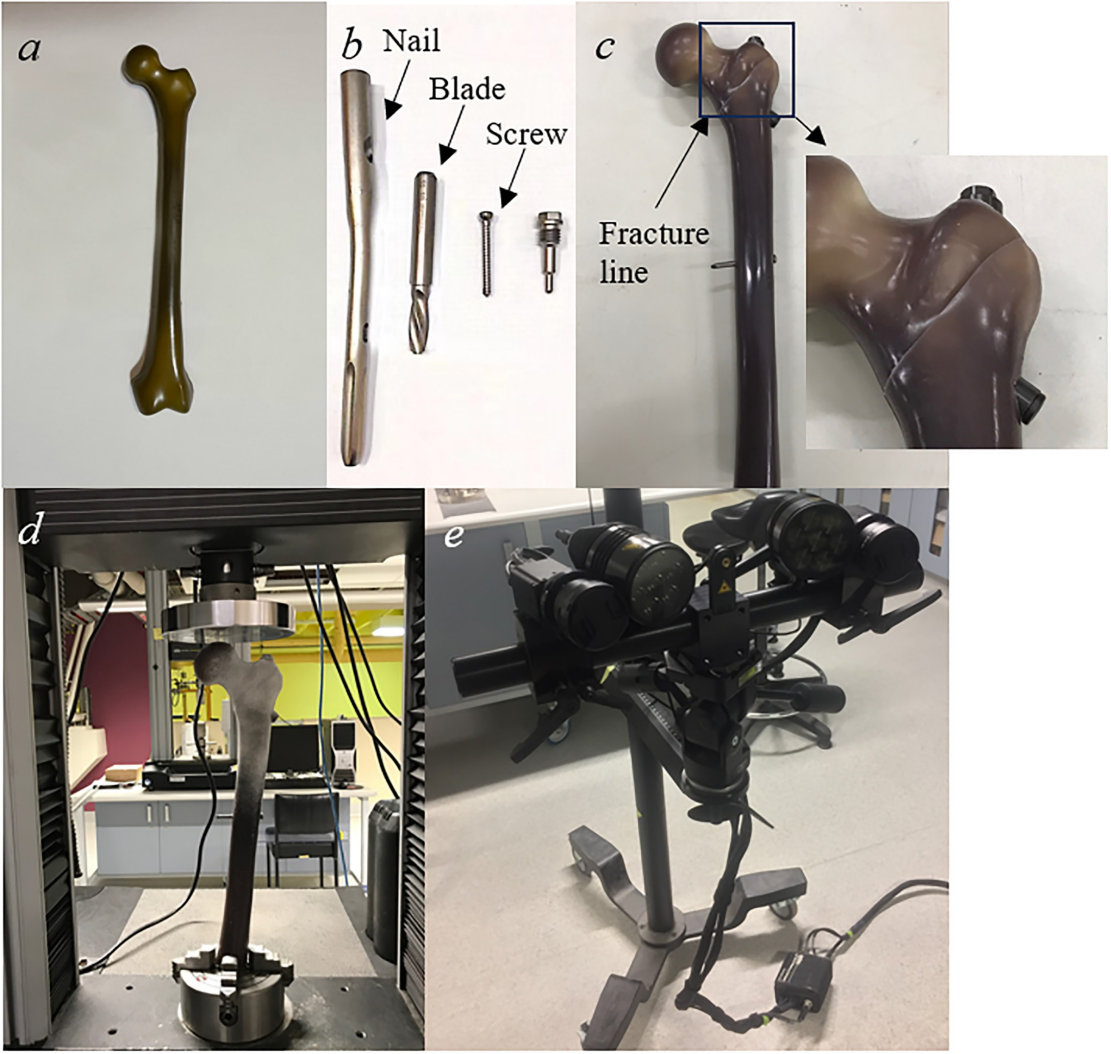
Mechanical testing of the (a) composite femur, (b) PFNA, (c) composite femur stabilized with PFNA, (d) INSTRON machine, (e) 3D optical ARMIS system.

Five 4^th^ generation composite femurs (Sawbones, Vashon, Washington, USA) were used in the experimental testing. These samples comprised glass-fiber reinforced epoxy and polyurethane foam, which effectively mimicked the properties of cortical and trabecular bones, respectively (Fig 2a). First, a fracture line was created on each surrogate based on AO/OTA classification 31/A1 [42] to create two-part stable intertrochanteric fracture in which the fracture gap was made negligible by an experienced orthopaedic registrar. The implantation was performed under C-arm fluoroscopy control. Subsequently, titanium PFNA (Degebaier, Wuhan, China) was inserted to secure the fractured bone according to the manufacturer’s guidelines. The specifications of PFNA are summarized in Table 1 (Fig 2b and 2c).

**Table 1:** Specifications corresponding to PFNA.

	Length (mm)	Diameter (mm)	Proximal diameter (mm)	Distal diameter (mm)	Angle(°)
Spiral blade	100	10			
Nail	170		16	10	
Locking bolt	32	5			
Nail neck stem angle					130

The bone fracture samples were subjected to axial loading tests using an INSTRON 5569A machine (INSTRON, Canton, Massachusetts, USA) with the distal portion of a sample firmly secured using a lathe chuck. The length of the distal portion under the lathe chuck was about 5 cm. Compressive loadings under different rates (*i*.*e*., 150N/s, 300N/s, 450N/s, 600N/s and 750N/s) were applied to the top of the femur head at the angle 10° of adduction and 9° of posterior flexion for simulating loading conditions during ambulation [22, 24, 43, 44]. Finally, an advanced three-dimensional (3D) optical measurement system—ARMIS (GOM, Braunschweig, Germany) which requires a speckle pattern to be applied to the surface of the fracture region of the composite femur was used to determine the vertical displacement of the femoral head based on the relative movement of the speckle pattern between images which were taken by high-speed camera (Fig 2e).

### Computational modeling of intertrochanteric fracture treated with PFNA

A 3D model of the femur, featuring a stable intertrochanteric fracture and a PFNA implant, was obtained using a CT machine (GE Healthcare, USA) that delivered an effective slice thickness of 1 mm, slice spacing of 0.625 mm and a pixel resolution of 512 × 512. The obtained 2D CT data, stored in DICOM format, were processed using Mimics software (version 21.0; Materialize, Leuven, Belgium). After the actions of region growth, mask editing, wrapping and smoothing in Mimics, the preliminary three-dimensional (3D) model was created and then imported into Geomagic Studio software (Raindrop Inc., Santa Clara, CA, USA). This process creates an optimized 3D model by surface optimization to eliminate computational instability for further modelling. The cortical and trabecular bones were separated using a 2 mm cortical layer reference from Treece et al. [45]. The fracture line, as discussed in the previous section, was integrated into the 3D numerical model.

**Fig 3:**
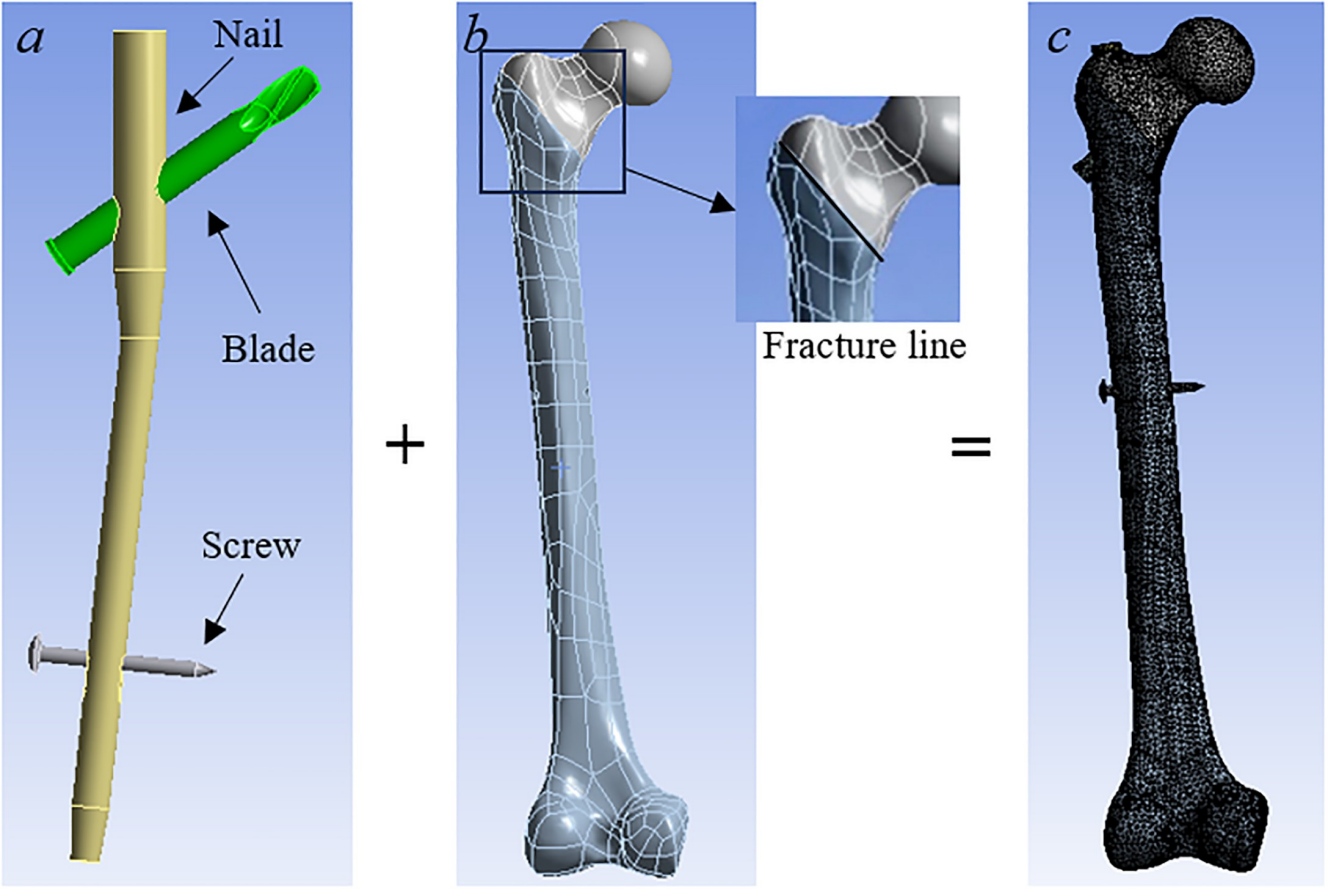
Development of integrated numerical model involving (a) PFNA, (b) femur, and (c) the complete model, respectively.

The 3D PFNA implant structure was constructed using SolidWorks (Dassault Systems, Velizy-Villacoublay, France) according to the manufacturer’s PFNA specifications. The assembly of the fractured femur and implant was executed within the same platform and subsequently linked to Ansys 16.0 (Ansys Inc., Canonsburg, PA) for all the following finite element analyses considered in this study. The elements of the model, including the cortical bone, trabecular bone, and PFNA, were meshed using 69,089, 54,373, and 20,548 4-noded tetrahedral elements, respectively. It has been pointed out by Fraldi et al. that tetrahedral elements are the most suitable elements for modelling biological tissues due to their abilities to handle complex shapes and geometries [46]. It is worthwhile to note that more advanced 10-noded tetrahedral elements are believed to provide a faster convergence rate and better outcomes [47]. Future studies should consider 10-noded tetrahedral elements. A frictional contact algorithm was used for the numerical analysis with frictional coefficients of 0.46, 0.23, and 0.30 for bone-bone interactions [48], implant–implant interactions [48], and bone-implant interactions [49], respectively. The contact interface between cortical and trabecular bones was considered as perfectly bonded. The distal end of the femoral model was rigidly secured, and quasi-static loading was implemented at the position indicated in the mechanical test (Fig 3). The contact simulation was performed in ANSYS based on augmented Lagrangian algorithm (ALA). A mesh convergence analysis was conducted to ensure precision. An average mesh size of 3 mm was set as the starting point, then mesh size with 0.5mm decreasing was applied for repeated simulations until the variation between the current simulated peak femur head displacement and subsequent one was less than 2%. A final average size of 2mm was considered to be the optimal size.

The elastic modulus and Poisson ratio of the implant are 113.8 GPa and 0.34, respectively. Both the cortical and trabecular bones were assumed to be linearly elastic and were calculated using the following equations [18, 27]:

$$E_{c}=-13450+14261\rho_{c}$$ (1)

$$E_{t}=6950\rho_{t}^{1.49}$$ (2)

where *E*_*c*_ and *E*_*t*_ (MPa) denote the elastic moduli of the cortical and trabecular bones, respectively. Variables *ρ*_*c*_ and *ρ*_*t*_ (in g/cm^3^) are the apparent bone mineral density of cortical bone and trabecular bone, respectively. The apparent BMD of cortical bone is approximately 1.86 g/cm^3^ [23], which gives an elastic modulus of 13095 MPa according to Eq (1). In addition, a Poisson ratio of 0.3 was assigned to the bone model.

This study primarily focused on determining the likelihood of mechanical failure of the trabecular bone under physiological loading. Therefore, the apparent BMD of the trabecular bone was represented as a normal distribution rather than as a distinct dataset. According to a study conducted by Nobakhti and Shefelbine who reviewed the relation between apparent BMD and elastic modulus of healthy and pathologic bones, the apparent BMD of trabecular bone for proximal femur mainly lay in the range of 0.09–0.9 g/cm^3^ [19]. Therefore, the mean value of apparent BMD of trabecular bone was assumed as the average of the lower and upper bonds. On the other hand, the standard deviation value assumed in this study was made in accordance with published data which gave a range of 20% -35% of mean value. Thus, a conservative value (*i*.*e*., 35% of the mean value) was assumed and applied to this study [50–52]. Based on these assumption, the mean and standard deviation of trabecular bone density were approximately 0.5 ± 0.175 g/cm^3^. Post-operative activity levels, quantified in terms of body weight (BW), are vital parameters. Assuming an average BW of 75 kg, the loading value would be approximately 750 N [25]. To encapsulate the post-operative mechanical loading impacting the proximal femur in daily exercises, the mean and standard deviation of physiological loading are posited at roughly 2 ± 0.4 BWs [53]. Latin hypercube sampling was used to estimate the distribution of apparent BMD and loading. This sophisticated sampling approach ensures faster convergence than conventional sampling methods, such as Monte Carlo simulations [54] (Sampling distribution plots for both BMD and BW are presented in S1 File).

### Mechanical failure criterion of the trabecular bone

The most frequent type of mechanical failure associated with intramedullary devices is cut-out [11]. While the trabecular bone can retain a significant proportion of its load-bearing capacity after yielding, strain yielding often signals the onset of mechanical instability owing to a loss in stiffness and strength [55]. Notably, a higher mechanical loading-induced strain was invariably correlated with a higher cut-out risk. Moreover, strain-based failure criteria are considered superior to stress-based criteria, as they provide a more comprehensive picture of tissue failure without being affected by inter-specimen differences in the apparent BMD [55]. Thus, in this study, the mechanical failure criterion for trabecular bone was determined using a compressive yielding strain of 0.85 ± 0.10% [50].

### Investigating the mechanical stability of intertrochanteric fracture treated with PFNA using engineering reliability analysis

In conventional engineering reliability analyses, the failure probability of a particular structure is described as the likelihood of the demand exceeding its capacity [34, 56, 57]. When considering the mechanical stability of an intertrochanteric fracture treated with PFNA, the probability of failure can be understood as the likelihood of the compressive strain induced by physiological loading in the proximal trabecular bone exceeding the compressive yielding strain. If the compressive strain of trabecular bone is denoted as ’*S*,’ and the compressive yielding strain as ’*Y*,’ the probability of failure (PoF) can be defined as follows:

$$PoF=P\left(S>Y\right)=P\left(Y-S<0\right)=P\left(Z<0\right)$$ (3)

where *Z* is the performance function.

The mean and standard deviation of *Z* were estimated using the following equations:

$$\mu_{Z}=\mu_{Y}-\mu_{S}$$ (4)

$$\sigma_{Z}=\sqrt{\sigma_{S}^{2}+\sigma_{Y}^{2}}$$ (5)

To calculate *μ*_*s*_ and *σ*_*s*_, the probability density function of *S* expressed in terms of random variables is proposed as follows:

$$S=g\left(X_{1},X_{2},\ldots \ldots X_{n}\right)$$ (6)

where *X*_*n*_ represents the random input variables associated with the apparent BMD and loading. The first-order Taylor series expansion [56, 58] is used to approximate the mean and standard deviation values of *S*:

$$\mu_{S}\approx g\left(\mu_{X_{1},}\mu_{X_{2},}\ldots \ldots \mu_{X_{n},}\right)$$ (7)

$$\sigma_{S}\approx \sqrt{\sum_{i=1}^{n}\sum_{j=1}^{n}\frac{dg}{dX_{i}}\frac{dg}{dX_{j}}COV\left(X_{i},X_{j}\right)}$$ (8)

where *μ*_*Xi*_ is the mean value of *X*_*i*_ and coefficient of variation (COV) (*X*_*i*_, *X*_*j*_) is the coefficient of variation of *X*_*i*_ and *X*_*j*_.

If the performance function is linear and the random variables are independent. The expression of *S*, its mean, and the standard deviation are given by Eqs (9)–(11), respectively:

$$S=a_{0}+a_{1}X_{1}+a_{2}X_{2}+\cdots a_{n}X_{n}$$ (9)

$$\mu_{S}=a_{0}+a_{1}\mu_{X_{1}}+a_{2}\mu_{X_{2}}+\cdots a_{n}\mu_{X_{n}}$$ (10)

$$\sigma_{S}=\sqrt{{\left(a_{1}\sigma_{X_{1}}\right)}^{2}+{\left(a_{2}\sigma_{X_{2}}\right)}^{2}+\cdots {\left(a_{n}\sigma_{X_{n}}\right)}^{2}}$$ (11)

In this study, a nonlinear multivariate model is proposed for *S* as below:

$${S=a}_{0}+a_{1}\times BMD+a_{2}\times BW+a_{3}BMD\times BW+a_{4}\times {BMD}^{2}+a_{5}\times {BW}^{2}$$ (12)

where BMD is the apparent bone mineral density, and BW is the loading expressed as the body weight.

To solve the nonlinear functions, Eqs (13)–(17) were used to estimate the mean and standard deviations of the nonlinear terms in Eq (12).

For *μ*_*p*_ and *σ*_*p*_ of the product of two random variables *X*_1_ and *X*_2_ [59].


$$\mu_{p}=\mu_{X_{1}}\mu_{X_{2}}$$ (13)

$$\sigma_{p}=\sqrt{\left(\sigma_{X_{1}}^{2}+\mu_{X_{1}}^{2}\right)\left(\sigma_{X_{2}}^{2}+\mu_{X_{2}}^{2}\right)-\mu_{X_{1}}^{2}\mu_{X_{2}}^{2}}$$ (14)

For *μ*_*q*_ and *σ*_*q*_ of *X*^2^.


$$\mu_{q}=\mu_{X}^{2}+\sigma_{X}^{2}$$ (15)

$$\sigma_{q}=\sqrt{4\mu_{X}^{2}\sigma_{X}^{2}+4\mu \gamma \sigma_{X}^{3}+\left(\kappa -1\right)\sigma_{X}^{4}}$$ (16)

If random variable *X* is normally distributed, the skewness and kurtosis of *X* are 0 and 3, respectively. Eq (14) can be simplified as:

$$\sigma_{q}=\sqrt{4\mu_{X}^{2}\sigma_{X}^{2}+2\sigma_{X}^{4}}$$ (17)

After linearizing the performance function, the unknown coefficients (*a*_1_, *a*_2_… *a*_5_), in Eq (10), can be estimated using regression analysis. Finally, the PoF can be estimated directly from:

$$PoF=1-\Phi \left(\frac{\mu_{Z}}{\sigma_{Z}}\right)$$ (18)

where Φ is the standard normal cumulative distribution function.

### Effect of age and daily activity on the mechanical failure of trabecular bone

This study assessed individuals aged > 50 years. The age groups separation (*i*.*e*., 50–64 years, 65–79 years, and ≥ 80 years) was made consistent with the one found in We divided patients into three age groups: 50–64 years, 65–79 years, and ≥ 80 years. Zebaze et al.’s clinical study in which the bone loss of cortical and trabecular bone is quantified for different age groups [60]. Utilizing measurements of hydroxyapatite in the proximal femur [60], and assuming that 40% of the trabecular bone is composed of hydroxyapatite [61], the mean BMD values for the three age groups are summarized in Table 2. We incorporated five daily activities into our analysis: normal walking, ascending stairs, descending stairs, standing up, and sitting down. The mean BW values were derived from a study by Bergmann et al. [53] and are detailed in Table 3. The standard deviation was calculated using the formula *σ* = *μ* × *COV*, where COV stands for the coefficient of variation. We chose a broad range of COV values (*i*.*e*., 0.1–0.9) to assess the effects of parameter uncertainty on the PoF.

**Table 2:** The mean and coefficient of variation (COV) of apparent bone mineral density (BMD) for different age groups [60, 6,1].

	50–64 years	65–79 years	≥80 years
Mean (g/cm^3^)	0.3375	0.2875	0.275
COV	0.1–0.9	0.1–0.9	0.1–0.9

Note: The mean BMD values were calculated according to the assumption that 40% of the trabecular bone is hydroxyapatite [61].

**Table 3:** The mean and coefficient of variation (COV) of physiological loading (expressed in terms of body weight, BW) for different activities of daily living [5,3].

	Normal walking	Up stairs	Down stairs	Standing up	Sitting down
Mean (BW)	2.38	2.51	2.60	1.90	1.56
COV	0.1–0.9	0.1–0.9	0.1–0.9	0.1–0.9	0.1–0.9

Note: it is assumed that one body weight is approximately 750 N [25].

## Results

**Fig 4:**
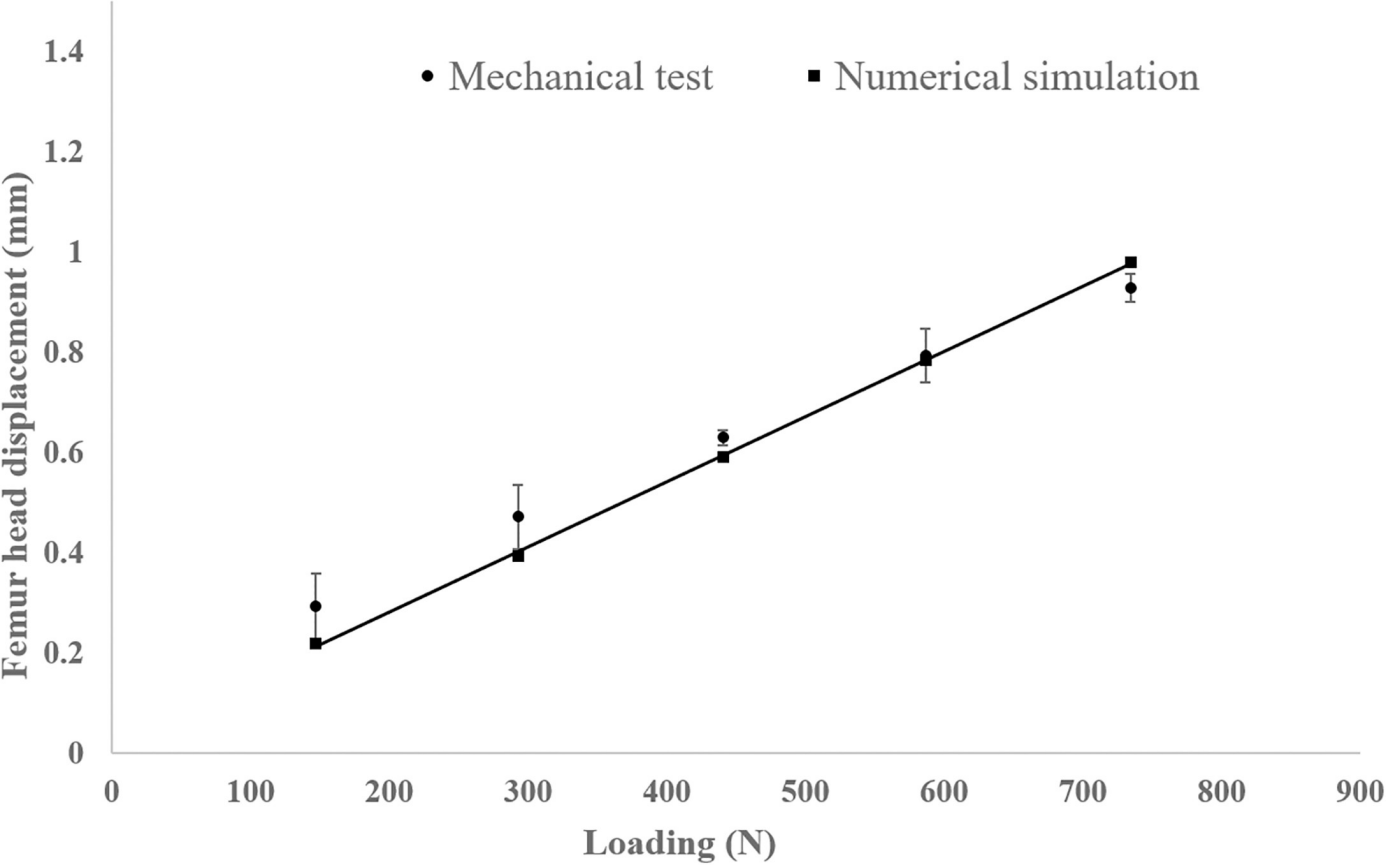
Comparison of the numerically predicted femur head displacement under different axial loads with experimental testing results.

The load-displacement curve derived from the numerical model was cross-validated against experimental findings. As shown in Fig 4, the numerical results show good agreement with the experimental data, with an average disparity between the two datasets of approximately 10%. Once validated, the numerical model is used to generate training data to estimate the nonlinear regression model of *S*. A series of 100 simulations was conducted in accordance with the mean and standard deviation values of BMD and BW recommended in previous section, in conjunction with the Latin hypercube sampling technique. By leveraging the results of these simulations and employing a least-squares curve-fitting method, an expression for *S* in relation to BMD and BW was obtained as follows:

$$S=10^{-3}\times \left(4.09-17.66\times BMD+2.80\times BW-2.35BMD\times BW+15.67\times {BMD}^{2}-0.05\times {BW}^{2}\right)$$ (19)

where the sum of the squared estimates of errors (SSE) = 4.316e-5, R-squared = 0.9334, adjusted R-squared = 0.9249, and root mean squared error (RMSE) = 0.0011.

**Fig 5:**
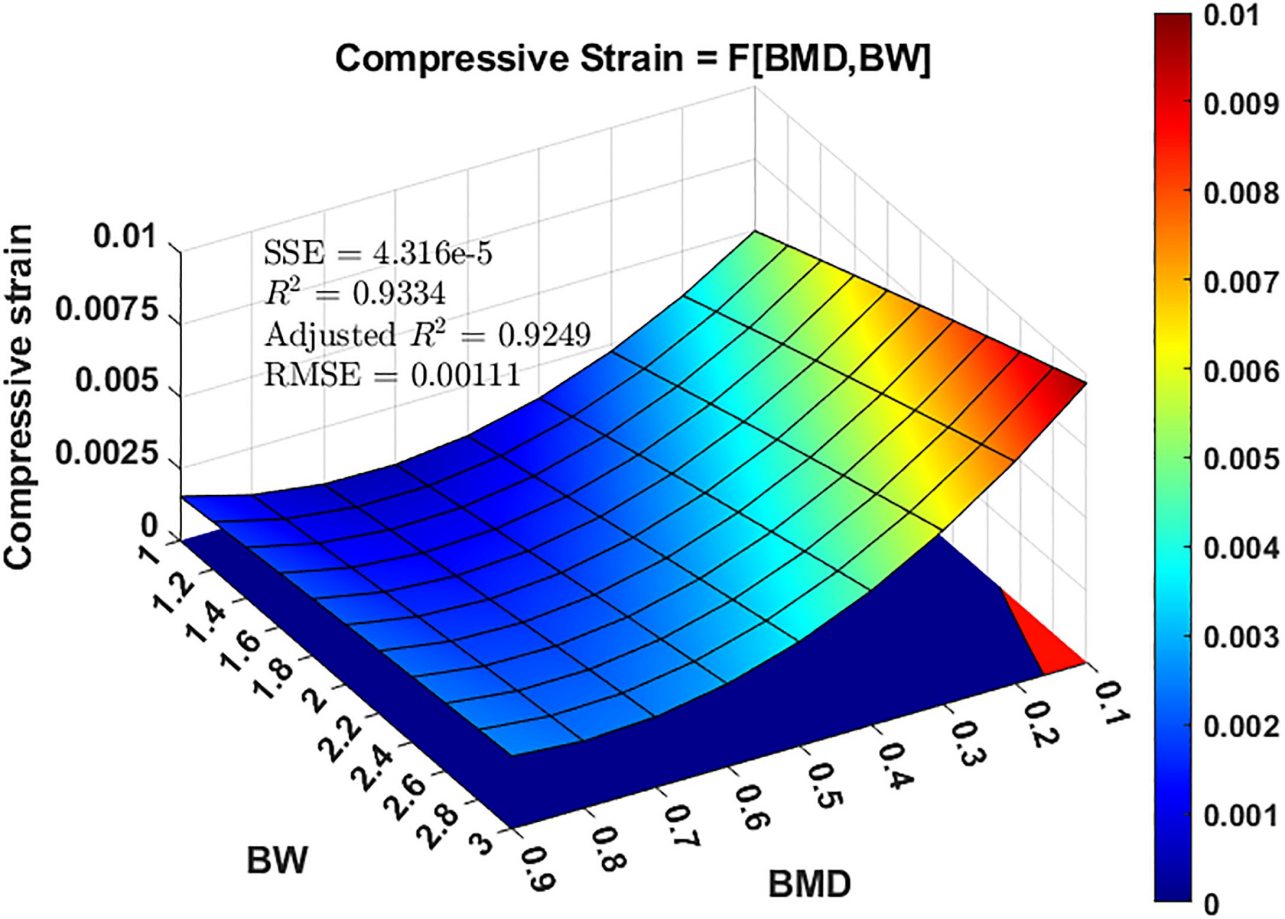
The relationship between loading (number of body weight) with apparent bone mineral density (BMD) and compressive strain at the proximal trabecular bone. The area on the x–y plane is the projection of 3D surface plot. Based on a compressive yielding strain value of 0.85%, the area highlighted in blue indicates the safe region, while the red area represents failure.

Fig 5 shows the interaction effects of BMD and BW on the compressive strain *S*. This shows that BW exhibited a positive linear relationship with *S*, whereas BMD displayed a negative nonlinear relationship with *S*. As the value of BMD increases, its impact on the compressive strain diminishes, reaching near convergence at approximately BMD = 0.6 g/cm^3^. Conversely, the effect of BW on the compressive strain remained relatively stable. Fig 5 further demonstrates how this information can be used to ascertain the threshold values of BW and BMD associated with trabecular bone failure, providing an illustrative example. The *x*–*y* plane projection of the 3D curve was divided into two regions based on the compressive yielding strain value of 0.85%. The blue-colored region represents the safe zone, and the red-colored area represents the failure zone. In this scenario, the failure region corresponds to BMD ≤ 0.15 g/cm^3^ and BW ≥ 2.5 approximately.

**Fig 6:**
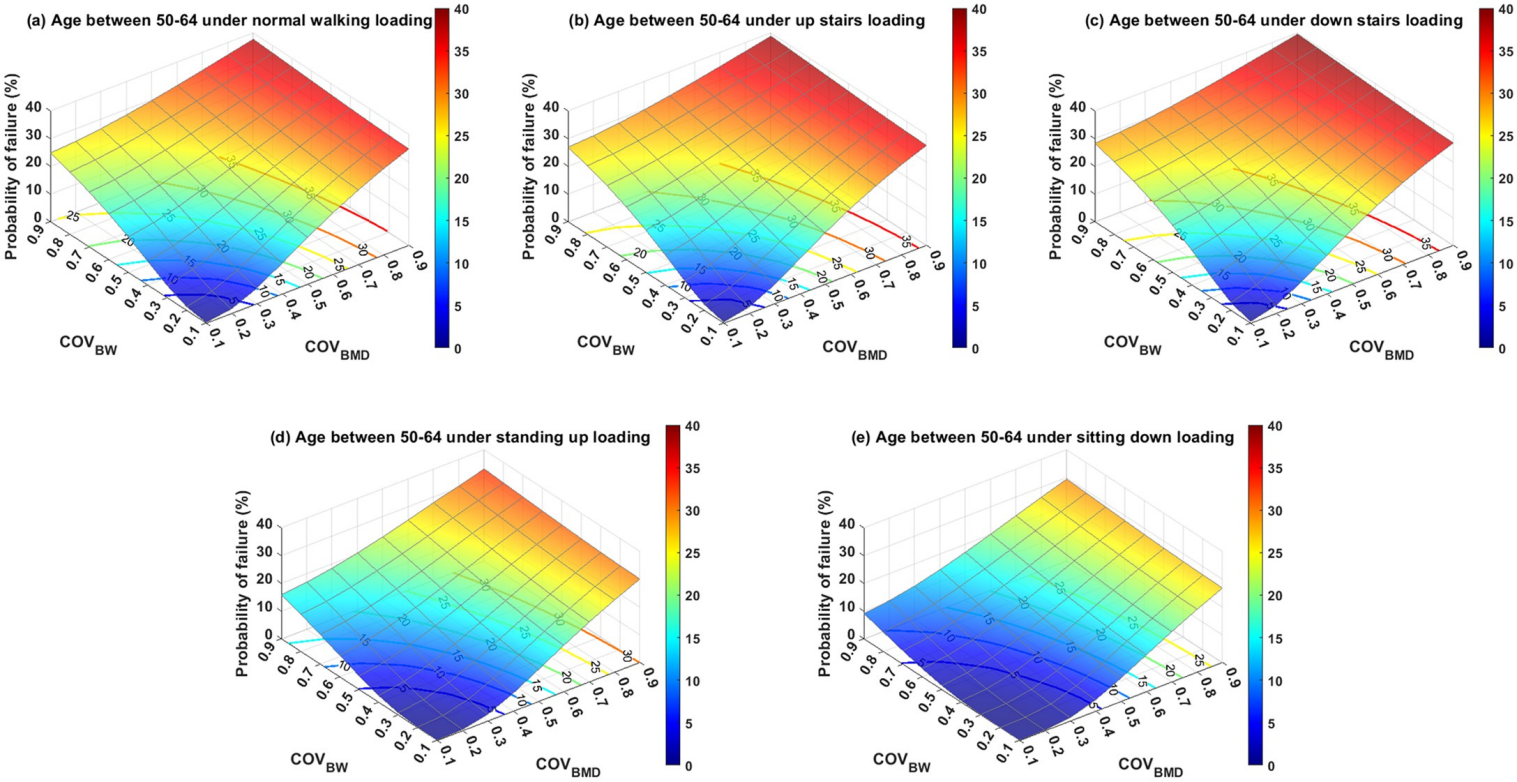
The probability of failure for patients aged 50–64 years who were subjected to loading induced by (a) normal walking, (b) walking up stairs, (c) walking down stairs, (d) standing up, and (e) sitting down. The 2D contour on the x–y plane is the cut-off line of the labeled probability of failure with respect to the 3D surface plot.

**Fig 7:**
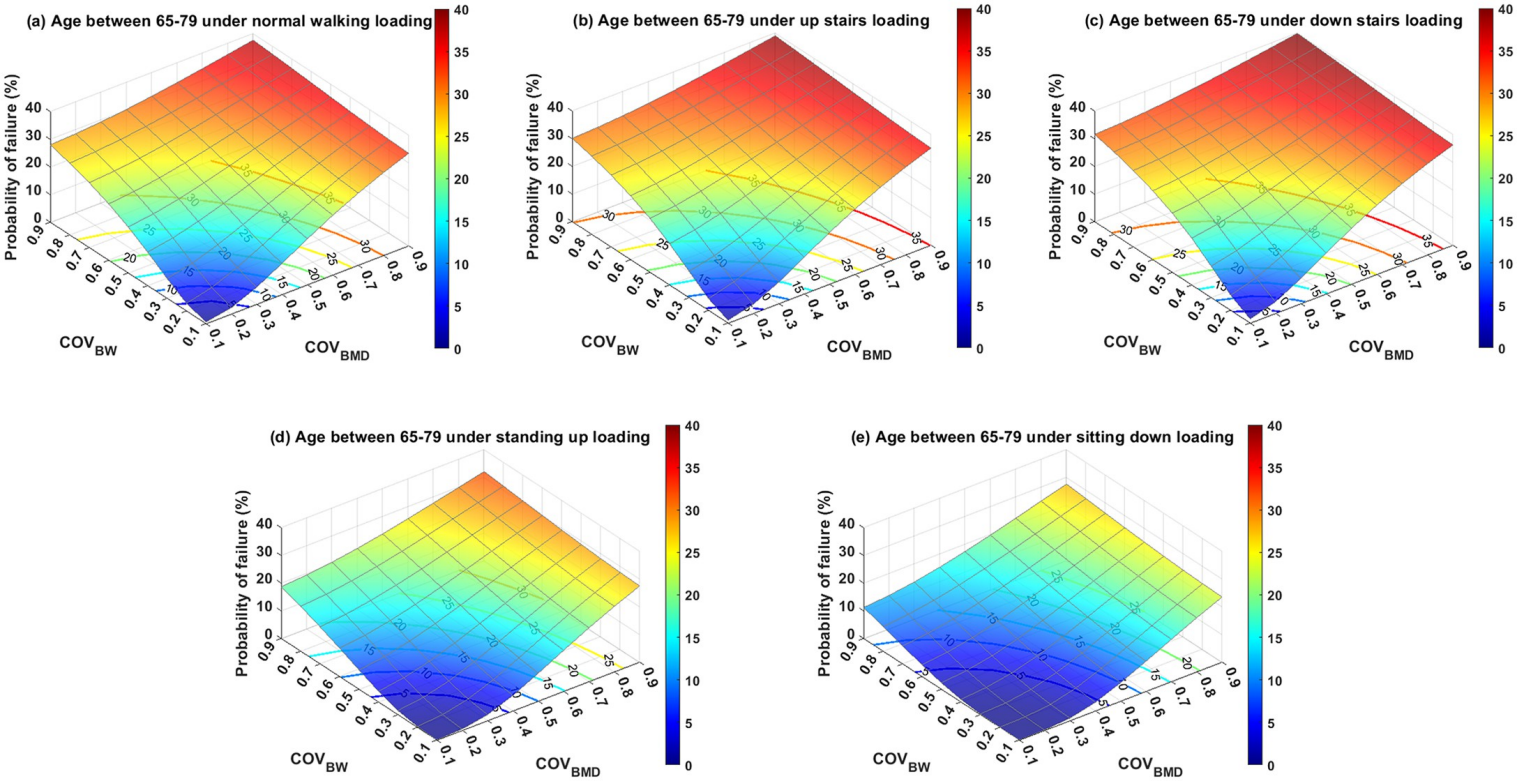
The probability of failure for patients aged 65–79 years who were subjected to loading induced by (a) normal walking, (b) walking up stairs, (c) walking down stairs, (d) standing up, and (e) sitting down. The 2D contour on the x–y plane is the cut-off line of the labeled probability of failure with respect to the 3D surface plot.

**Fig 8:**
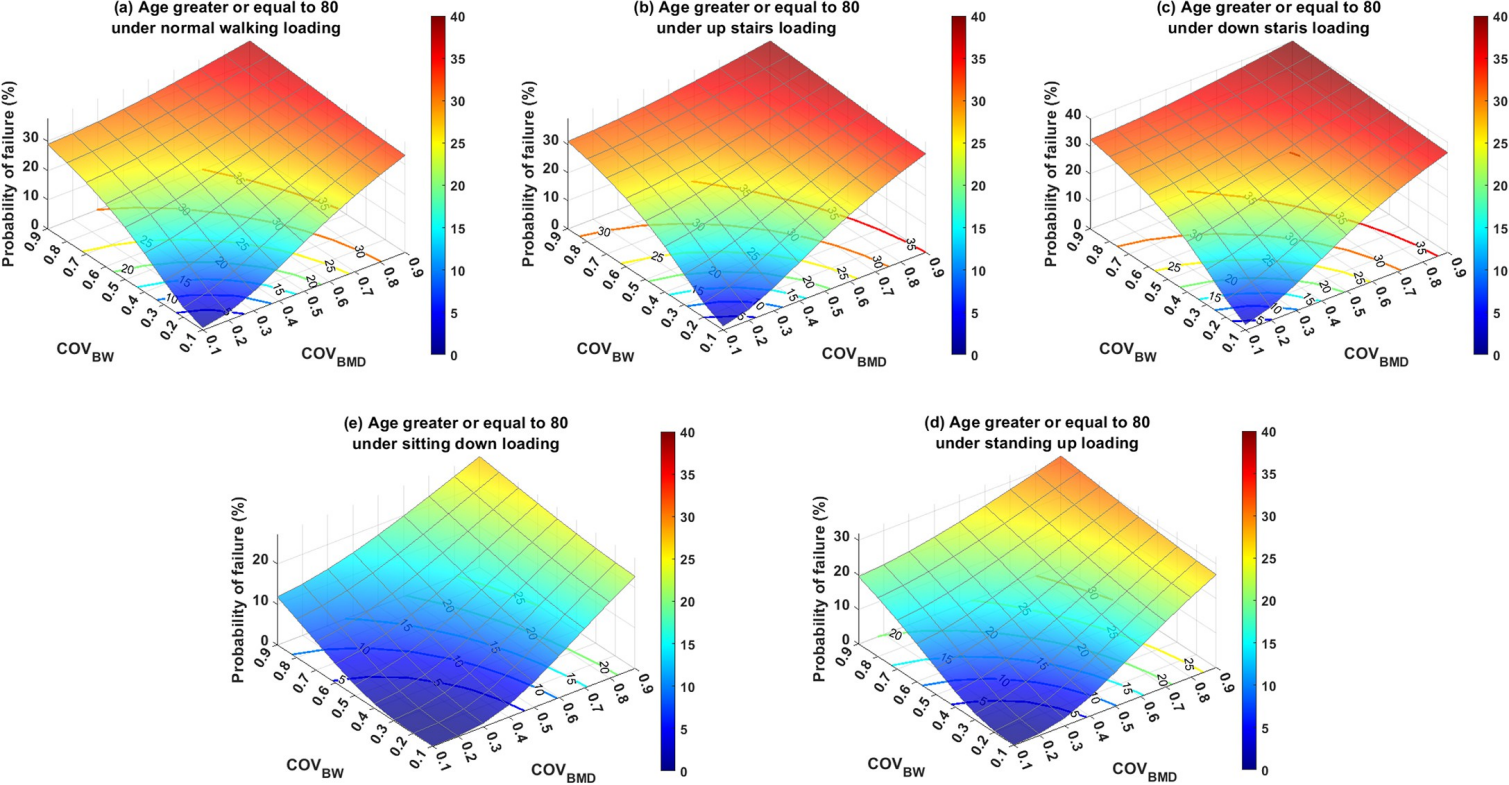
The probability of failure for patients aged ≥ 80 years who were subjected to loading induced by (a) normal walking, (b) walking up stairs, (c) walking down stairs, (d) standing up, and (e) sitting down. The 2D contour on the x–y plane is the cut-off line of the labeled probability of failure with respect to the 3D surface plot.

Subsequent analyses evaluated the PoF in relation to age and everyday activities, utilizing their respective mean and coefficient of variation (COV) values, as reported in Tables 2 and 3. To facilitate comparisons, the PoFs for various scenarios were projected onto their x–y planes and represented as 2D contours labeled with PoF levels, (Figs 6–8). Fig 6 specifically represents the PoF for patients aged 50–64 who suffer from intertrochanteric fractures while engaging in five distinct daily activities. There appeared to be a nonlinear correlation between the COV of BMD (COV_BMD_) and PoF. Interestingly, COV of BW (COV_BW_) exhibits a nonlinear relationship with PoF when COV_BW_ ≤ 0.4, and a linear relationship when COV_BW_ > 0.4. Furthermore, variation in BW significantly affected PoF when BMD variation was low for all activities under consideration. However, this impact decreases when the BMD variation is high. For example, under a normal walking load on the fractured bone and a COV_BMD_ of 0.1, the PoF increased from 0.18% to 24.79% as COV_BW_ increases from 0.1 to 0.9. In contrast, when the same loading condition was used and the COV_BMD_ increased from 0.1 to 0.9, the PoF only increased from 34.79% to 37.81%. Similarly, the variation in BMD, when combined with low COV_BW_, had a more substantial influence on PoF than when paired with high COV_BW_. Overall, the older age groups required relatively fewer variations in BMD and BW to reach a particular PoF level than the younger groups. This implies that younger patients demonstrate a greater tolerance to uncertainties in BMD and BW.

Regarding the assessment of activities, it was found that non-ambulatory actions, such as standing up and sitting down, were relatively safe for all age groups, provided that the variation in BW was effectively managed (*i*.*e*., COV_BW_ was relatively low). However, ambulatory activities involving walking, specifically inclined and declined walking (*i*.*e*., ascending and descending stairs), can result in a PoF value exceeding 5% if the standard deviation of the BW surpasses 20% of its mean for individuals aged over 65 years. Among all the scenarios assessed in this study, the highest PoF was estimated to be approximately 40.17%. This occurred when a patient with COVs of both BMD and BW of 0.9 descended stairs. Conversely, the PoF approached zero when a patient performed actions such as standing up or sitting down with both COV_BMD_ and COV_BW_ equal to 0.1.

## Discussion

With the increasing life expectancy, the incidence rate of intertrochanteric fractures is expected to increase. Surgical treatment using internal fixation, such as PFNA, has gained popularity due to its superior biomechanical stability compared to extramedullary fixation. However, post-operative complications, such as cut-out due to biomechanical instability, remain a significant concern for both surgeons and physiotherapists, as evidenced by several clinical studies [62–64]. Cut-out is often associated with the strain level of the trabecular bone. Compressive yielding strain has been widely used to assess the risk of cut-out [22, 23]. However, most of these studies relied on deterministic analyses, which neglected the uncertainties associated with the mechanical properties of the trabecular bone and the load applied to the fractured bone. Goldstein et al.’s research on the mechanical properties of human trabecular bone revealed that the variation in Young’s modulus could be up to 100-fold within a single specimen [65]. Moreover, the experimental methodology, loading rate, and loading direction significantly contribute to the variation in the elastic modulus of the trabecular bone [19]. In contrast, Vasarhelyi et al. conducted clinical trials to assess the precision of partial weight-bearing in patients with lower-extremity fractures. Their clinical findings demonstrated that none of the patients achieved the prescribed level of PWB [66]. The questionable accuracy of postsurgical weight-bearing significantly contributes to the variation in mechanical loading acting on the fractured bone; the variation was as high as 119% [66].

This study employed an engineering reliability technique in conjunction with numerical modeling to evaluate the PoF under various combinations of trabecular bone conditions and physiological loadings. PoF was defined as the likelihood of the strain in the proximal femoral trabecular bone induced by mechanical loading, exceeding its compressive yielding strain. It is widely recognized that mechanical instability often occurs after strain yielding [55]. Both of our numerical and experimental studies indicated that the amount of loading acting on the femur head (*i*.*e*., BW) featured a strong positive correlation with the peak micromotion of the fractured femur. Similar outcome can be found in Viceconti et al.’s study in which a statistical finite element model in conjunction with experimental method were developed to evaluate the primary stability of a cementless hip stem. Classical Monte Carlo scheme with Latin Hypercube sampling technique was applied in their study for statistical parameterization [41]. A finite element model for intertrochanteric fractures was developed and validated using mechanical tests. Using this numerical model, along with the Latin hypercube technique, we proposed a multivariate regression model for compressive strain (*S*) in relation to BMD and BW. The results revealed strong correlations between the compressive strain *S* and the magnitudes of BMD and BW. As expected, high BMD and low BW values typically result in relatively small compressive strains. However, when BMD exceeded 0.6g/cm^3^, the influence of BMD on compressive strain became negligible.

The application of regression model *S* is depicted in Fig 5, wherein the safe and failure regions are differentiated by color based on a compressive strain value of 0.85%. A combination of BMD ≤ 0.15 g/cm^3^ and BW ≥ 2.5 may potentially lead to trabecular bone failure under compression. Referring to a clinical study conducted by Cummings et al., the mean BMD for 83 Caucasian women, aged ≥ 65 with hip fractures, was found to be 0.17 g/cm^3^ [67]. A subsequent study by Center et al. reported a slightly higher value (0.25 g/cm^3^), based on the BMD measurements of 73 women with hip fractures [68]. In terms of the five daily activities examined in this study, only ascending and descending stairs (with BW of 2.51 and 2.60, respectively) generated hip contact loading that surpassed the threshold value of BW = 2.5. With regards to lower limb fracture rehabilitation, Schwachmeyer et al. analyzed the peak hip contact loading for 13 common physiotherapeutic exercises, ranging from weight-bearing to dynamic exercises. Their findings highlighted that normal walking resulted in a loading of 2.66 BW, whereas one-leg bridging and standing on the injured leg produced a loading value of 3.03 BW. All other exercises remained below 2.5 BW [69]. Thus, the clinical data indicate that most daily living and rehabilitation activities are safe for patients of any age. However, it is important to note that the question of mechanical failure in the trabecular bone is not a simple binary "yes or no.” Uncertainties associated with loading on the fractured bone, material properties of the trabecular bone, and failure criteria (compressive yielding strain in this study) should all be considered in decision-making.

To further investigate the impact of uncertainties related to age and activities on PoF, a supplementary analysis was conducted. This study examined three age groups (50–64 years old, 65–79 years old, and 80 years) and five common activities of daily living (normal walking, ascending stairs, descending stairs, standing up, and sitting down). The uncertainties in these variables were represented by their COV, where a higher COV value indicated a higher level of uncertainty. The COV range in our study varied from 0.1 to 0.9, to encompass outcomes from the most optimistic to the most pessimistic scenarios. The results revealed that PoF was highly sensitive to variations in BMD. Given that the mean BMD of the 65–79 age group was similar to that of the 80 years and older groups, the uncertainties of BMD and BW for both age groups showed a similar correlation with PoF. Drawing on experimental data from three separate studies [50, 51, 70], we found that the average standard deviation of BMD was approximately 25% of its mean value, which corresponds to COV_BMD_ = 0.25. Assuming a PoF threshold of 5% as an acceptable regime, the predicted PoF results suggest that individuals aged > 65 years should avoid ascending stairs, while those aged > 50 years should be cautious while descending stairs. Another significant source of uncertainty affecting PoF is physiological loading. Patients with hip fractures often struggle to adhere to the post-operative weight-bearing regimens established by orthopedists or physiotherapists. Our simulation results underscore that the PoF is particularly sensitive to variations in loading when the uncertainty in the BMD is low. Conversely, the effect of BW variation on the PoF decreased when the uncertainty in the BMD was high. These findings suggest that variations in BMD play a significant role in influencing PoF.

As illustrated in Figs 6–8, less variation in the assessed variables results in a lower PoF. Thus, the accuracy of BMD measurements and enhancement of patient compliance with weight-bearing therapy are of paramount importance. To enhance weight-bearing compliance, biofeedback devices have emerged as effective tools. Several such devices are currently available to improve weight-bearing compliance. For instance, Sensistep sandals [71], OpenGo Science insoles [72], and SmartStep insoles [73] have been clinically validated to monitor and assist patients with lower-extremity fractures in adhering to prescribed full or partial weight-bearing regimens. However, large-scale clinical studies are necessary to evaluate the long-term benefits. The determination of trabecular bone material properties presents a significant challenge owing to the complex architecture of the bone. Plate and end-cap techniques are frequently employed in experimental setups to ascertain the material properties of the trabecular bone. Helgason et al. discussed the advantages and disadvantages of the two experimental methods [17]. Although methodological discrepancies often account for variations in the trabecular bone material properties, Linde et al. stated that the geometry of the trabecular bone specimen is another crucial factor [74]. The influence of the anatomical site on material properties has also been documented in numerous studies [50]. Therefore, careful selection of trabecular bone specimens, attention to specimen geometry, and judicious selection of experimental setups are crucial for reducing measurement variability.

This study had some limitations that merit attention. First, the mathematical relationship between the elastic modulus and apparent BMD was drawn from the existing literature. Given the impact of the experimental method, specimen geometry, and anatomical site on the material properties of the cortical and trabecular bones, the selected mathematical model may not be the most appropriate for this investigation. Future studies should conduct compression tests on bone specimens acquired from specific anatomical sites. Second, only BW and BMD of trabecular bone have been analyzed in this study, however, there are some other factors, such as apparent density of cortical bone, femur size, regions of bone cavity in contact with the implant and types of loading also play important roles in mechanical stability of damaged femur after treatment. For example, Courtney et al. analyzed the effects of age on post-yield damage in cortical bone. Their study suggested that older cortical bone has higher chance to develop microcrack at a given loading level [75]. Dopico-Gonzalez et al. evaluated the effect of femur characteristics on mechanical performance of total hip replacement. Their study concluded that uncertainty and variability of both femur and implant geometry should be incorporated into the probabilistic analysis of the uncemented THR [37]. The effect of impact loading induced by sideway falls on the mechanical performance of extramedullary fixation implants was evaluated by Nag et al. [76]. Third, this study utilized a linear elastic material model to characterize the trabecular bone, which may not fully represent its true mechanical behavior. A poroelastic model comprising solid and liquid phases may offer a better depiction of the mechanical behavior of cancellous bone [77]. Therefore, future studies should consider implementing the theory of porous media. In addition, this study did not consider muscle loading. Future research should aim to develop a musculoskeletal model that includes muscle loading to provide a more accurate representation. Finally, instead of regression analysis, artificial neural networks (ANN) can be employed because they have been demonstrated to offer superior estimations [78, 79]. Future studies should consider implementing this advanced analytical method to improve the results. Although this study focuses on the mechanical performance of stable intertrochanteric fracture stabilized with PFNA, it is still worthwhile to note that fatigue loading also plays an important role in the mechanical performance of implants [80]. Once the material of implant is weakened by a cycling loading, localized crack is initiated, then followed by crack propagation, and finally reaching ultimate failure [81]. Hence it is recommended to looking into fatigue failure of PFNA in future studies.

## Conclusion

In this study, a probabilistic model was developed using finite element modeling and engineering reliability techniques to predict the failure probability of trabecular bones under physiological loading. The effects of age and activity type on this probability were evaluated. The simulation results suggest that patients over 50 years of age who have experienced intertrochanteric fractures should avoid activities such as descending stairs during post-operative treatment with PFNA. Additionally, those aged > 65 years should avoid ascending stairs. These findings highlight the importance of reducing uncertainties associated with the mechanical properties of the trabecular bone and physiological loadings to significantly reduce the probability of trabecular bone failure. These results have the potential to inform post-operative rehabilitation guidelines for patients with intertrochanteric fractures. However, before implementing these findings in the development of rehabilitation strategies, the limitations of this study must be addressed. Future research should focus on testing more accurate mathematical models, using more representative material models, considering muscle loadings, and utilizing advanced estimation techniques, such as ANN, to enhance accuracy and effectiveness.

## Supporting information

S1 File Sampling distribution plots for both BMD and BW. (DOCX)
