# Supplementary material for: A probabilistic approach for assessing the mechanical performance of intertrochanteric fracture stabilized with proximal femoral nail antirotation
Source: PLoS One. 2024 Apr 11;19(4):e0299996. doi: 10.1371/journal.pone.0299996 (PMC11008846; doi:10.1371/journal.pone.0299996)
Supplement: S1 File — (DOCX) [file pone.0299996.s001.docx]

The cumulative distribution function (CDF) plots of both BMD and BW

Fig 1. Sampling distribution of bone mineral density of trabecular bone (BMD)

Fig 2. Sampling distribution of loading expressed in terms of body weight (BW)
